# The European Parliament proposal for the new EU General Data Protection Regulation may severely restrict European epidemiological research

**DOI:** 10.1007/s10654-014-9909-0

**Published:** 2014-05-07

**Authors:** Olof Nyrén, Magnus Stenbeck, Henrik Grönberg

**Affiliations:** 1 Department of Medical Epidemiology and Biostatistics, Karolinska Institutet, 171 77 Stockholm, Sweden; 2 Division for Insurance Medicine, Department of Clinical Neuroscience, Karolinska Institutet, 171 77 Stockholm, Sweden

In January 2012, the European Commission presented the draft of a new General Data Protection Regulation (GDPR) to the European Parliament and the Council of the European Union. The GDPR is planned to replace the 1995 Directive 95/46/EC, which constitutes the present European legal framework for processing of personal data. Hence, this new binding Regulation will lay the legal foundation for future European epidemiology based on personal data, including register-based research.

The intentions behind the new GDPR are commendable: [1] to protect the fundamental rights and freedoms of individuals, in particular their right to protection of personal data, in a society where commercial enterprises and authorities have rapidly increasing capabilities to collect, store and combine personal information; and [2] to facilitate free movement of personal data within the European Union through a uniform legislation in all member states.

The Commission’s proposal is being reviewed and amended independently by the Council of the European Union and the European Parliament. In the Parliament, the Committee on Civil Liberties, Justice and Home Affairs (LIBE) was assigned the task of formulating the Parliament’s amendments. The first draft by the chairman of the Committee, Jan Philipp Albrecht, was criticized for insufficient consideration to the needs of epidemiological research. The proposed text threatened to restrict currently existing possibilities to produce scientific evidence based on European data analysis and, in turn, to impede efforts to improve public health and welfare in the union and elsewhere.

In October 2013, after a long period of negotiations surrounded by intense lobbying efforts, the LIBE Committee voted on its final amendments to the Commission’s proposal [1]. Alas, although some improvements were noted, the overall outcome was largely disappointing from an epidemiological perspective. The main points are summarized in the following.

The first Articles with specific relevance for scientific research are concerned with general principles (Article 5) and lawfulness (Article 6) of personal data processing. Article 5b lays down that personal data shall be collected for specified, explicit and legitimate purposes and may not be further processed in a way incompatible with those purposes (“purpose limitation”). This corresponds to an identical principle in the current 95/46/EC Directive. However, in Directive 95/46/EC there was an exemption for research, namely that further processing of data for historical, statistical or scientific purposes is not to be considered as incompatible with the original purpose as long as Member States provide appropriate safeguards. This exemption was omitted in LIBE’s amendments, dramatically reducing the scope for data sharing between research groups and severely restraining the use of retrospective (historic) cohort study designs. Such studies utilize old data collections with exposure information that was collected for other purposes than the current scientific research. Thus, hundreds of thousands person-years of follow-up may have accumulated already at the start of the retrospective cohort study, making it possible to immediately test important public health hypotheses that would otherwise take decades to address. A typical example is the study of long-term health effects of Swedish snus (snuff) in an already existing cohort of construction workers [2]. If taken literally, the omission of the exemption threatens to eliminate the possibility to use administrative registers for epidemiological research altogether.

## Articles 6.2 and 83: shaky pillars forming the legal foundation

Article 6 establishes the necessary prerequisites for any lawful processing of personal data. In its second paragraph (6.2) it lays down that processing “which is necessary for the purposes of historical, statistical or scientific research” is lawful as long as the processing adheres to the provisions given in Article 83. Article 83, however, is remarkably meagre; all it says is that processing of personal data for the purposes of historical, statistical or scientific research is allowed only if these purposes cannot be otherwise fulfilled using anonymous data and that “data enabling the attribution of information to an identified or identifiable data subject is kept separately from the other information under the highest technical standards, and all necessary measures are taken to prevent unwarranted re-identification of the data subjects” (i.e., pseudonymisation is mandatory). Of note, Article 83 does not mention informed consent among its conditions.

Another paragraph (6.1) in Article 6 states that processing shall be lawful ***only*** if at least one of six specified conditions (a-f) is met. Consent is one such condition, whereas scientific research is not. Our interpretation is that 6.2 overrides 6.1 and that Article 6 approves processing of personal data for scientific research purposes, even in the absence of consent. However, Article 6 might be interpreted differently by others. If so, obligatory consent will be required for all research using personal data, including epidemiological research.

A general problem with the Article 6.2–83 axis is that while it implies that the relevant conditions in the two Articles will fully determine the scope for the processing of personal data for historical, statistical or scientific research purposes, derogations for research appears in Articles 5e, 9.2i, 17.3c, and 81.2. These scattered single derogations imply that all other parts of the Regulation are meant to be applicable to scientific research. This generates confusion and may create unintended impediments for research.

## An amendment to Article 81 is a serious threat to large-scale epidemiological research

Article 9.2i lays down that processing of ***sensitive*** personal data, including data concerning health, is allowed when necessary for historical, statistical and scientific research purposes subject to the conditions referred to in Article 83. However, a very unfortunate amendment by LIBE to Article 81, dealing with processing of data concerning health, notably for the due operation of health care services, has materially disturbed the original apparent symmetry between Articles 6, 9, 81, and 83. The revised Article 81.2 says that “processing of personal data concerning health which is necessary for historical, statistical or scientific research purposes shall be permitted ***only with the consent of the data subject***, and shall be subject to the conditions and safeguards referred to in Article 83”. The additional stipulation of mandatory informed consent makes the cross-reference between 6.2 and 83 somewhat misleading.

Admittedly, an accompanying amendment (81.2a) and Recitals 123 and 123a open for the possibility that Member States law may provide for exceptions to the requirement of consent, with regard to research that serves a high public interest. Then, in addition to obligatory pseudonymisation and with reference to Article 19, the data subjects are explicitly given the right to object at any time. That Article 19 only concerns processing based on points (d) and (e) of Article 6.1, not on processing for scientific research purposes according to Article 6.2, further emphasizes the anomalous character of Article 81.2a. What constitutes “high public interest” is to be determined by the Commission via delegated acts, after consultations with the European Data Protection Board. This is indeed an important encroachment on the subsidiarity principle of the European Union.

The legal practice following from these provisions—if enacted without further changes—remains conjectural, but a restrictive interpretation may have devastating effects on large-scale epidemiological research where collection of informed consent is unfeasible, or where non-participation threaten to bias the results. Such studies constitute a significant part of the combined European epidemiological literature; one example of the former is the Swedish-Danish SCANDAT blood donation and transfusion database covering donations, transfusions, and long-term health outcomes among 1.1 million donors and 1.3 million recipients as far back as 1966 [3]. With approvals from the ethics boards, the data were derived from computerised administrative blood bank databases and high-quality health registers, allowing precise estimations of disease concordance among donors and recipients indicative of possible transmission of diseases such as cancer, Alzheimer’s and Parkinson’s diseases [4]. Another excellent example of a study sensitive to bias caused by refusal or inability to obtain informed consent is a British study measuring the cancer risk among almost 180,000 persons who underwent CT scans in childhood, in order to develop guidelines for safe use of CT scans in clinical practice [5].

An additional drawback of the reliance on Member State law for exemption from the obligatory consent will be that the intended uniformity of research-related legislation throughout Europe will not be attained, maintaining existing obstacles for free movement of research data across European borders.

## Uncertainties about the future of health registers

The status of the high-quality health registers—epidemiological crown jewels for public health statistics and public health policy in several European countries and essentially indispensable resources in health research by virtue of their completeness and virtual absence of bias—might become a cliffhanger. The registers will first stumble on the previously mentioned LIBE amendment requiring consent (Article 81.2), and then remain at the mercy of national legislation, which may or may not waive the obligatory consent but cannot remove the obligatory pseudonymisation or the right for the data subjects to object. Moreover, even if national laws will support the collection of personal data on health without consent of the data subjects, the release of health register data for research conducted by other researchers may require consent.

## Mandatory pseudonymisation: not a trivial issue

Obligatory pseudonymisation (data enabling identification of specific data subjects being kept separately from the other information) might be seen as a small and reasonable concession, but if strictly interpreted the consequences for epidemiological research may be detrimental. In the present LIBE amendment, personal data is defined as data that contains a unique personal identifier (direct identification) or data that can be attributed to a person without the presence of an identifier because of the richness of the available information. The combination of a few key variables (e.g., age, sex, date of diagnosis, geographic region, and diagnosis code) in a contingency table often results in some cells with just a single observation, providing a possibility for indirect identification of at least some subjects. If indirect identification is to be counted as “data enabling attribution of information to a data subject”, then research databases must be stripped of considerable amounts of information in order to adhere to the requirement of pseudonymisation, possibly rendering many—if not most of them—useless for epidemiological research.

In addition, as convincingly argued previously [6] pseudonymisation is likely to be influenced by trivial errors in the data used in the pseudonymisation process. This will increase the risk of missed linkages of data on single individuals. Even if these error rates are small, a simulation has indicated that the effect on aggregated measures such as e.g. survival may be far from trivial. Therefore, strict adherence to the pseudonymisation rule will likely result in a general loss of quality of data in existing health registers. There are no explicit provisions regarding the lawfulness of, or procedures for, warranted re-identification for the purpose of e.g. record linkages, quality control of data, or verification of conducted research. The mere acknowledgement of the existence of a key file, and the retained exemptions for research data from the data storage minimization rule (no longer than necessary for the purpose) in Article 5e and from the data subject’s right to erasure of data in Article 17.3c and Recital 53, however, lead us to believe that re-identification, when necessary, will be lawful. It would be helpful if Article 83 would explicitly state that the pseudonymisation requirement can be lawfully waived during checking or matching operations and also acknowledge that processing of identifiable personal data is sometimes necessary for sustaining the highest quality in epidemiological research.

## What next?

While the aim was to complete this legislative process before the Parliament election in May 2014, it has now become apparent to all parties that the goal will not be attained. In order to avoid having to start from scratch again after the election, the Parliament endorsed LIBE’s amendments to the Regulation with 621 votes in favour, 10 against and 22 abstentions in a plenary voting on March 12, 2014. Although this strong support underscores the gravity of the situation for European epidemiology and register-based research, the battle is not yet lost. The Council of the European Union—the other part of the essentially bicameral EU legislature—needs to agree on a position. In order for the legislation to become a reality, the wordings of the Parliament and the Council texts have to agree exactly. The current aim of The Working Party on Information Exchange and Data Protection (DAPIX), which handles the review of the Regulation in the Council, is to present a draft to the Minister meeting in June 2014, but more realistically DAPIX needs another 3–6 months to finish its work. Thereafter, a “second reading” process will ensue, in which the Council and the Parliament negotiate a final draft. Thus, there are still opportunities to ensure that the Council adopts a more research-friendly position which averts the imminent threats to large-scale epidemiologic studies and register-based research in general. It must be acknowledged that the view on integrity issues differs between European Member States, based on historical experiences and long-term tradition. Moreover, while there is a broad consensus that the protection of individuals’ personal data should be strengthened when technical developments open endless opportunities to collect and combine such information, the willingness to put trust in the scientific community and entrust scientists with exemptions varies. The LIBE amendment represents a hardline stand with only few concessions specifically for research, adapted to Member States with the least favourable conditions for large-scale epidemiology, but admittedly with some option for Member States to relax the provisions. We believe that a more fruitful approach would be to try to adapt to existing research-related legislation in Member States with the most developed large-scale epidemiology. This legislation appears to have struck a balance between the citizens’ legitimate wish to preserve their integrity and public health interests, notably the requisites for truly valid health-related research, with unspoiled trust among the public and essentially no examples of important misbehaviour on the part of the scientific community. We propose that the following suggestions are forwarded:

1. As pointed out in amendment proposals from the European Parliament’s Committee on Industry, Research and Energy and Committee on Legal Affairs, an exemption from the purpose limitation in Article 5(b), corresponding to the existing exemption in the current Directive 95/46/EC, should be reintroduced.
2. Article 81.2 should be removed entirely. Then, 81.2a becomes obsolete.
3. The pseudonymization requirement in Article 83 needs to be relaxed. Pseudonymized data should be defined as data where the direct identifier is kept separately from the other information, and should not be extended to indirect identification. The need for re-identification to attain precise linkages, data verification and quality control must be accommodated.
4. Restore the “6.2–83 axis”. Article 6 ought to be revised so that it becomes clear that 6.2 (establishing the lawfulness of processing of personal data for the purposes of historical, statistical or scientific research) overrules 6.1. Other provisions relevant to scientific research (exemption from the “storage minimization principle” in 5e, exemption from the prohibition against processing of sensitive data in 9.2i, exemption from the “right to erasure” in 17.3c, and the hopefully reintroduced exemption from “purpose limitation”) should be moved to Article 83. There, the text must clearly convey that where exemptions are made, Article 83 overrules the provisions from which scientific research is being exempted.

Epidemiologists and other researchers throughout Europe should use their contact networks to put pressure on their respective governments to act via the Council of the European Union and on their representatives in the European Parliament so that European public health research is rescued before it is too late.
